# *PTEN* mRNA expression is less pronounced in left- than right-sided colon cancer: a retrospective observational study

**DOI:** 10.1186/s12885-016-2400-4

**Published:** 2016-06-13

**Authors:** Hidekazu Kuramochi, Ayako Nakamura, Go Nakajima, Yuka Kaneko, Tatsuo Araida, Masakazu Yamamoto, Kazuhiko Hayashi

**Affiliations:** Department of Chemotherapy and Palliative Care, Tokyo Women’s Medical University, 8-1 Kawadacho, Shinjuku-ku, Tokyo, Japan; Department of Surgery, Tokyo Women’s Medical University, Yachiyo Medical Center, 477-96 Owadashinden, Yachiyoshi, Chiba, 276-8524 Japan; Department of Gastrointestinal Surgery, Tokyo Women’s Medical University, 8-1 Kawadacho, Shinjuku-ku, Tokyo, Japan

**Keywords:** Colorectal cancer, Signaling pathway, Primary tumor location, PTEN, Gene expression, Liver metastases

## Abstract

**Background:**

Several recent studies have reported that patients with metastatic colorectal cancer (CRC) whose primary tumor is located in left side of the colon have more favorable responses to anti-epidermal growth factor receptor (EGFR) antibody therapy than those with right-sided tumors. However, the mechanism for this phenomenon is unknown.

**Methods:**

Fifty-two cases of primary CRC with liver metastases were analyzed in this retrospective study. The mRNA levels of 19 signal transduction genes in both primary tumor and liver metastases were measured by real-time reverse transcription polymerase chain reaction. The purposes of this study were (1) to determine the correspondence between signal transduction gene expressions in primary tumors and corresponding liver metastases, and (2) to determine whether expression levels of these genes differ by primary tumor location.

**Results:**

mRNA expression levels of 14 of 19 signal transduction genes, including *PTEN*, *ERBB2, MET, HGF, AREG,* and *EREG*, showed significant correlations between the primary tumor and corresponding liver metastases. When the mRNA levels of the primary tumors were compared by tumor location, only *PTEN* mRNA expression differed significantly between left and right-sided CRC (median PTEN expression: left 1.00 vs. right 1.68; *p* = 0.017). When rectal cancers were separated from left-sided colon cancers, *PTEN* mRNA levels increased progressively from rectum to right-sided colon (median; rectum 0.84, left colon 1.23, right colon 1.68, *p* = 0.013). *PTEN* mRNA expression in liver metastases also differed significantly according to primary tumor location (median; left 0.92 vs. right 1.27, *p* = 0.048). There was no difference in overall survival between patients with high versus low levels of *PTEN* mRNA (*p* = 0.59).

**Conclusions:**

Our data suggest that the PIK3/AKT/mTOR pathway is more active in left- than right-sided CRC, which provides a possible explanation for the fact that efficacy of anti-EGFR therapy differs by location of primary tumor.

## Background

Recent progress in chemotherapy for metastatic colorectal cancer (mCRC) has been remarkable, with many novel drugs including molecular targeted agents having been developed. Anti-epidermal growth factor receptor (EGFR) antibody drugs, such as cetuximab and panitumumab, often combined with cytotoxic drugs, are now widely used as front-line chemotherapy for Ras wild-type mCRC, and are demonstrating promising results [1, 2]. Several recently published studies have demonstrated an association between the effect of anti-EGFR antibody and the location of the primary tumor in patients with mCRC. Brule et al. reported that, in the NCIC CO.17 study, patients with left-sided tumors had significantly longer progression-free survival when treated with cetuximab compared with best supportive care, whereas those with right-sided did not [3]. Einem et al. reported that, in the AIO KRK-0104 trial, left-sided tumors were associated with significantly longer overall and progression-free survival than right-sided tumors among patients with KRAS wild-type CRC when treated with cetuximab as first-line treatment [4].

The left and right colon have different embryologic origins, the left colon being from the foregut and the right from the midgut, and different blood supplies. Therefore, as first proposed by Bufills et al. [5], left- and right-sided colon cancers are distinct genetic entities. However, no molecular biomarkers that elucidate the reason(s) for the differences in the effects of these agents according to tumor location have yet been identified.

Liver metastases are the main cause of death in most patients with mCRC. Because controlling liver metastases is considered essential in the treatment of mCRC, it is reasonable to expect that the level of mRNA expression of molecular target genes in liver metastases would be the best predictor of therapy benefit. However, in many—if not most—cases, only biopsies of the patient’s primary tumor are readily available for analysis. Therefore, it is important to investigate the relationship between levels of response determinants in the primary tumor and in the corresponding liver metastases to determine whether analysis of biopsies of the primary tumor is useful for prediction of tumor response.

In this study, we have analyzed the mRNA expression levels of 19 signal transduction genes from both the primary tumors and corresponding liver metastases of 52 patients with CRC and resectable liver metastases. The purposes of this study were (1) to determine the relationship between signal transduction gene expressions in primary tumors and corresponding liver metastases, and (2) to determine whether expression levels of these genes differ by location of the primary tumor site in patients with mCRC.

## Methods

### Patients and samples

Data from 52 cases of primary CRC with liver metastases were analyzed in this retrospective study (35 men and 17 women; median age, 61.5 years (range, 32–91 years). The metastases were synchronous in 33 patients and metachronous in 19. The patients had undergone surgical resection of primary colorectal adenocarcinomas and liver metastases between 1995 and 2007 in the Department of Gastroenterology, Tokyo Women’s Medical University, Tokyo, Japan. All patients were Japanese and all had given their written informed consent according to the institutional regulations. Relevant characteristics of the 52 patients are shown in Table 1. They were classified as having right-sided CRC if the primary tumor was located in the cecum, ascending colon, hepatic flexure, or transverse colon, and left-sided CRC if the tumor site was within the splenic flexure, descending colon, sigmoid colon, or rectum.

**Table 1:** Patient Characteristics

Gender	M/F	35/17	Lymph node	pN0	16
Age	Median (range)	61.5 (32–91)		pN1,N2	36
			Location of primary tumor	rectum	15
Pathology	Well differentiated	40		left colon	20
	Moderate-poor	10		right colon	17
	Mucinous	2	Liver metastases	synchronous	33
Depth	pT2	1		metachronous	19
	pT3	7	KRAS	wild	26
	pT4	44	(codon12,13)	mutant	23

This study was approved by the Ethics Committee of Tokyo Women’s Medical University and performed in accordance with the Declaration of Helsinki.

### Microdissection

Formalin-fixed, paraffin-embedded tumor specimens were cut into 10 μm thick serial sections. For pathological diagnosis, one slide was stained with hematoxylin and eosin and evaluated by a pathologist. Manual microdissection using a scalpel was performed if the histology was homogeneous and contained more than 90 % cancer cells. For all other samples, laser-capture microdissection (P.A.L.M. Microlaser Technologies AG, Munich, Germany) was performed to ensure that only tumor cells were dissected.

### RNA isolation and cDNA synthesis

Isolation of RNA from formalin-fixed paraffin-embedded (FFPE) specimens was performed using an RNeasy FFPE Kit (Qiagen, Tokyo, Japan) according to the manufacturer’s instructions. cDNA was converted from the total RNA yielded, using a High Capacity cDNA Reverse Transcription Kit (Applied Biosystems, Tokyo, Japan).

### Reverse transcription-PCR

cDNA was pre-amplified using a Taqman PreAmp Master Mix Kit (Applied Biosystems) according to the manufacturer’s instructions. mRNA expression of 19 signal transduction genes and a single internal reference gene (beta-2-macrogloblin) were measured by a fluorescence-based real-time polymerase chain reaction (PCR) detection method (StepOne real-time PCR system, Applied Biosystems). A list of the 19 genes and primers/probes information for the Taqman Gene Expression Assays (Applied Biosystems) is provided in Table 2. The PCR reaction mixture consisted of 10 μL of Taqman Fast Universal PCR Master Mix, No UNG (Applied Biosystems), 5 μL of pre-amplified cDNA sample, 1 μL of Taqman Gene Expression Assays primers and probe (20×), and 3 μL of nuclease-free water. Cycling conditions were 95 °C for 20 s, followed by 40 cycles at 95 °C for 1 s and 60 °C for 20 s. The threshold cycle (CT) value for each gene was determined by SDS software v1.2 (Applied Biosystems). The delta-CT(ΔCT) value, which is the difference between the CT value of the target gene and that of the endogenous control gene, was calculated by the same software. Delta-ΔCT (ΔΔCT), which is the difference in the ΔCT value for each sample and the highest ΔCT value as a calibrator, was also calculated and the 2^-ΔΔCT^ number used for relative mRNA quantification.

**Table 2:** Target gene characteristics

Gene symbol	Gene name	Location	Primer No.
AREG	amphiregulin	4q13.3	Hs00950669_m1
BTC	betacellulin	4q13.3	Hs01101204_m1*
EGF	epidermal growth factor	4q25	Hs01099999_m1
EGFR	epidermal growth factor receptor	7p12	Hs00193306_m1
ERBB2	erb-b2 receptor tyrosine kinase 2	17q12	Hs01001580_m1*
ERBB3	erb-b2 receptor tyrosine kinase 3	12q13	Hs00176538_m1*
ERBB4	erb-b2 receptor tyrosine kinase 4	2q33.3-q34	Hs00955525_m1*
EREG	epiregulin	4q13.3	Hs00914312_m1
HBEGF	heparin-binding EGF-like growth factor	5q23	Hs00181813_m1*
HGF	hepatocyte growth factor	7q21.1	Hs00300159_m1*
IGF1	insulin-like growth factor 1	12q23.2	Hs01547656_m1*
IGF1R	insulin-like growth factor 1 receptor	15q26.3	Hs00609566_m1
IGF2	insulin-like growth factor 2	11p15.5	Hs01005962_m1
IGF2R	insulin-like growth factor 2 receptor	6q26	Hs00181419_m1*
MET	met proto-oncogene	7q31	Hs01565584_m1*
MST1	macrophage stimulating 1	3p21	Hs00360684_m1
MST1R	macrophage stimulating 1 receptor	3p21.3	Hs00899925_m1*
PTEN	phosphatase and tensin homolog	10q23.3	Hs02621230_s1
TGFA	transforming growth factor, alpha	2p13	Hs00608187_m1
B2M	beta 2-microglobulin	15q21-q22.2	Hs99999907_m1

Median values were used as the cut-off values to divide strong from weak expression.

### Screening for KRAS mutation

DNA was extracted from the FFPE specimens using a Qiamp DNA FFPE tissue Kit (Qiagen, Tokyo, Japan) according to the manufacturer’s instructions. KRAS codon 12,13 mutations were measured by direct-sequencing method, as previously described [6].

### Statistical analysis

Median mRNA levels for left- and the right-sided tumors, and PTEN mRNA levels by KRAS status were compared using the Wilcoxon signed-rank test. The correlation between the mRNA levels of primary tumors and corresponding liver metastases was assessed using Spearman’s rank correlation. KRAS mutation frequency was compared between left- and the right-sided tumors using Fisher’s exact test. The Kaplan–Meier method was used to construct survival curves and the log-rank test for statistical analysis. Overall survival was defined as the time from the day of primary tumor resection to death from any cause. Statistical analyses were performed using JMP 10 (SAS Institute, Cary, NC, USA). Statistical significance was recognized at P-values of less than 0.05. All values are two-sided.

## Results

### Comparison of mRNA expression in primary tumors and corresponding liver metastases

Correlations between mRNA expression levels of 19 genes in primary tumors and the corresponding liver metastases are shown in Table 3. Significant correlations were identified for *AREG* (*p* = 0.023), *EREG* (*p* = 0.0022), *EGF* (*p* = 0.0057), *EGFR* (*p* = 0.0007), *ERBB2* (also known as *HER2*) (*p* < 0.0001), *ERBB3* (*p* = 0.044), *HBEGF* (*p* = 0.014), *HGF* (*p* = 0.0023), *MET* (*p* = 0.0005), *IGF1R* (*p* < 0.0001), *IGF2R* (*p* < 0.0001), *MST1R* (*p* = 0.0069), *PTEN* (*p* = 0.0003), and *TGFA* (*p* < 0.0001), whereas no significant correlations were identified for *BTC, ERBB4, IGF, IGF2,* and *MST1.*

**Table 3:** Correlation between mRNA levels in primary tumors and liver

Gene symbol	Spearman rank-order correlation coefficient (Rs)	p-value
AREG	0.31	0.023*
EREG	0.42	0.0022*
BTC	0.16	0.27
EGF	0.38	0.0057*
EGFR	0.46	0.0007*
ERBB2	0.55	<0.0001*
ERBB3	0.28	0.044*
ERBB4	0.11	0.42
HBEGF	0.34	0.014*
HGF	0.41	0.0023*
MET	0.47	0.0005*
IGF	0.23	0.094
IGF1R	0.61	<0.0001*
IGF2	0.051	0.72
IGF2R	0.65	<0.0001*
MST1	0.054	0.70
MST1R	0.37	0.0069*
PTEN	0.48	0.0003*
TGFA	0.55	<0.0001*

* statistically significant (*p* < 0.05)

### Comparison of mRNA levels and KRAS status between left- and right-sided CRC

**Fig. 1:**
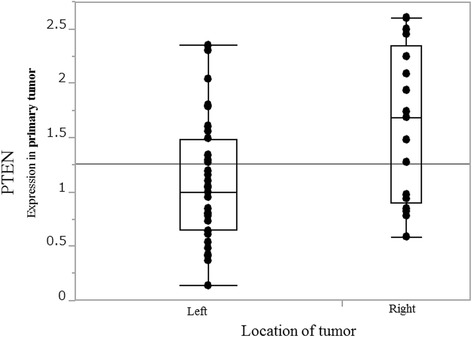
PTEN mRNA expression in primary tumor by tumor location PTEN mRNA levels are significantly higher in right- than in left-sided colorectal cancer (median; left 1.00 vs. right 1.68, *p* = 0.017)

**Fig. 2:**
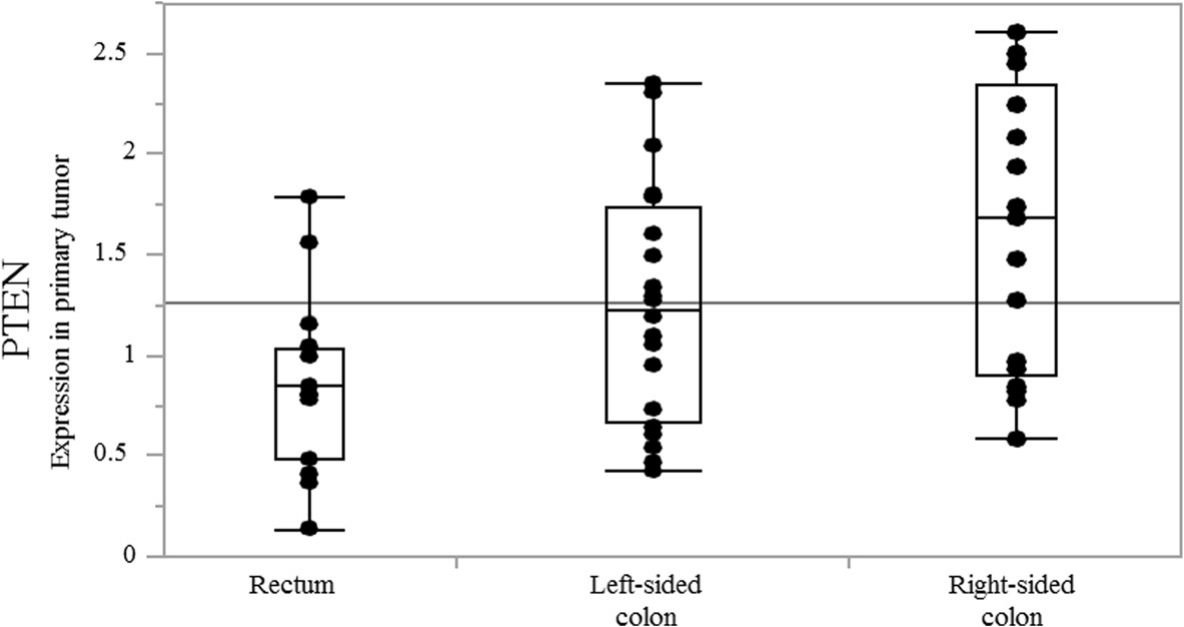
PTEN mRNA expression in primary tumor by location in rectum, left-sided colon, and right-sided colon *PTEN* mRNA levels progressively increase from rectum to right-sided colon (median; rectum 0.84, left colon 1.23, right colon 1.68, *p* = 0.013)

**Fig. 3:**
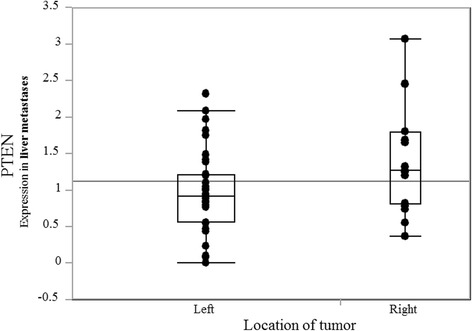
PTEN mRNA expression in liver metastases by location of primary tumor PTEN mRNA levels are significantly higher in liver metastases from right-sided colon cancer than in those from left-sided colorectal cancer (median; left 0.92 vs. right 1.27, *p* = 0.048)

Thirty-five patients were had left-sided and 17 right-sided CRCs. The primary tumor mRNA expression levels of 19 genes were compared between left- and right-sided cancers: only *PTEN* mRNA expression differed significantly (median; left 1.00 vs. right 1.68; *p* = 0.017) (Fig. 1). When rectal (*n* = 15) were separated from left-sided colon CRCs (*n* = 20), *PTEN* mRNA levels progressively increased from rectum to right-sided colon (median; rectum 0.84, left colon 1.23, right colon 1.68; *p* = 0.013) (Fig. 2). *PTEN* mRNA expression in liver metastases also differed significantly according to primary tumor location (median; left 0.92 vs. right 1.27; *p* = 0.048) (Fig. 3).

**Fig. 4:**
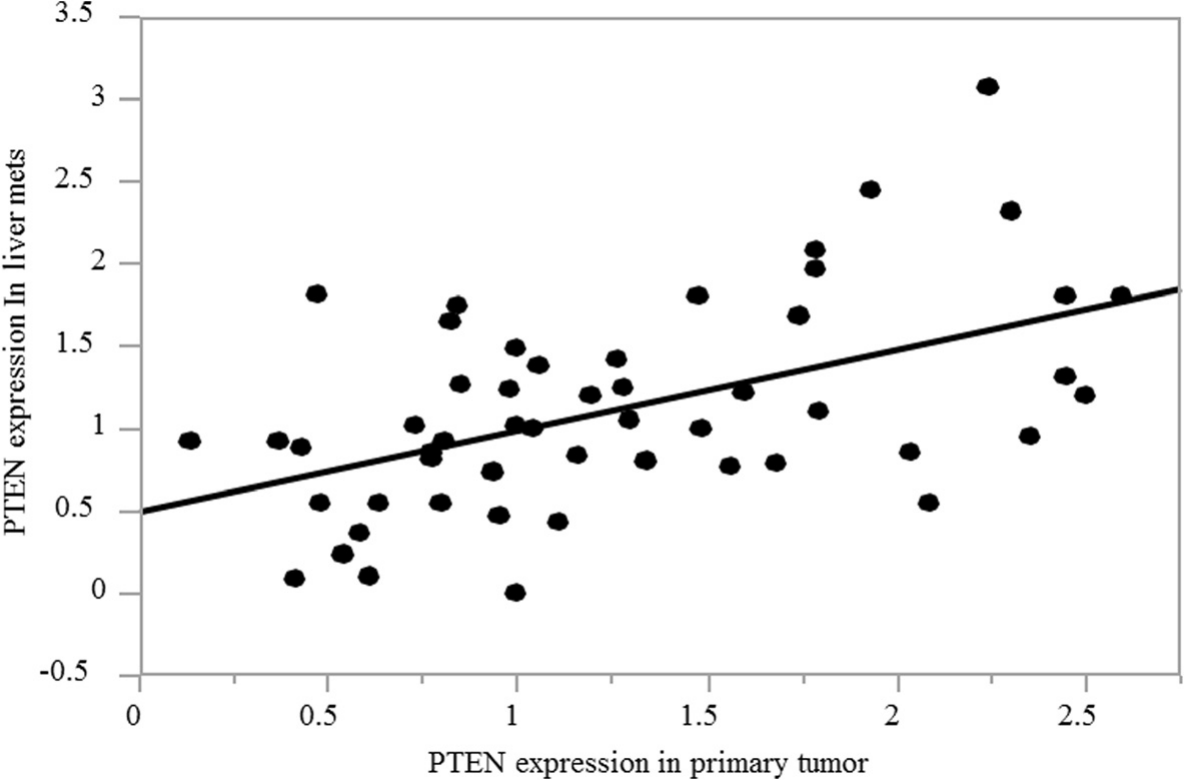
Correlation of PTEN mRNA expression between primary tumor and liver metastases. There is a significant correlation between PTEN mRNA expression in primary tumor and liver metastases. Spearman’s correlation coefficient 0.48 (*p* = 0.0003)

The correlation of PTEN mRNA expression in primary tumors and corresponding liver metastases is shown in Fig. 4. The Spearman correlation coefficient was 0.48 (*p* = 0.0003).

There was a non-significant tendency toward a higher frequency of KRAS codon 12,13 mutation in right-sided CRC (KRAS mutation %: left 37.5 % vs. right 64.7 %; *p* = 0.082).

Amounts of PTEN mRNA in the primary tumor did not differ significantly between patients with *KRAS* wild and mutant genes (PTEN median 1.22 vs. 1.00, *p* = 0.21).

### PTEN expression and overall survival

When the median value of *PTEN* mRNA levels in the primary tumor was used as a cut-off line, there was no difference in overall survival between patients with high and low levels of *PTEN* mRNA (*p* = 0.59).

## Discussion

Several recent several studies have reported that the location of CRCs is associated with response to anti-EGFR antibody [3, 4, 7, 8]. All these studies found that left-sided CRCs are associated with more favorable responses and prognosis than are right-sided. However, the mechanism(s) for this phenomenon has not been identified.

In this study, only *PTEN* mRNA levels differed significantly between left- and right-sided CRCs. *PTEN* mRNA levels progressively increased from rectum to right-sided colon. PTEN is a downstream inhibitor of the PI3K/Akt/mTOR pathway, one of the EGFR pathways, which modulates cell proliferation and survival [9]. Loss of the PTEN tumor suppressor function has been observed in various cancers, such as those of breast, prostate, thyroid, and endometrial origin [10]. Our finding of weaker *PTEN* expression in left- than right-sided side CRC indicates that the PI3K pathway is more active in left- than right-sided CRC. Johnson et al. reported that, in 154 patients with CRC, expression of PI3K/Akt/mTOR pathway components as measured by immunohistochemistry was stronger in left-than right-sided CRCs [11], which supports our data. If the PI3K-mTOR pathway is more active in left-sided lesions, it is unsurprising that blocking the EGFR pathway with anti-EGFR antibody seems to be more effective in left- than right-sided CRCs.

Although PTEN is known to have germline mutation and deletion by allelic loss [12–15], it has been suggested that PTEN may be inactivated by mechanisms other than mutations and/or deletions. Goel et al. reported that hypermethylation of the *PTEN* promoter correlates significantly with either decreased or complete loss of PTEN protein expression [10]. Several studies have found that PTEN loss and PIK3CA mutation are associated with resistance to anti-EGFR therapy [14, 16, 17]. However, others have reported that PTEN and PIK3CA mutation have no relationship with the outcome of anti-EGFR therapy [18, 19]. The usefulness of PIC3CA and PTEN as biomarkers is still controversial; they appear to be less reliable biomarkers than RAS or BRAF.

Mao et al. pooled data from eight studies and found a concordance rate of 71.7 % for PTEN expression in primary CRCs and their metastases [20]. This concordance rate is relatively small compared with those for *KRAS* mutation (92 %), *BRAF* mutation (96.8 %), and *PIK3CA* mutation (93.9 %). Although immunohistochemistry (IHC) was used in most of the PTEN studies included in this meta-analysis, in our study we used mRNA expression levels as measured by quantitative real-time reverse transcription polymerase chain reaction. This quantitative method showed a relatively strong correlation between primary CRCs and their liver metastases. Hocking et al. used Taqman copy number variation and IHC to assess loss of PTEN function in 51 patients with CRC and reported a concordance rate of 68 % between Taqman copy number and IHC in assessment of PTEN loss [21]. Reported findings seem to vary between methodologies: standardized assessment of PTEN function is required to clarify the role of PTEN as a biomarker.

We found significant correlations of mRNA expression between primary tumor and liver metastates for 14 genes, but not for the other five. A recent study showed that HER2- (ERBB2) positive CRC has favorable responses to trastuzumab, which is an anti-HER2 antibody [22]. Even when a resected primary tumor is HER2-positive, if HER2 expression differs between the primary and metastatic tumors, different strategies should be considered for treating the metastatic lesions. However, our data indicate a strong correlation (*p* < 0.0001) between mRNA expression of *ERBB2* in primary tumors and liver metastases, which suggests that anti-HER2 drugs can be used to treat metastases when the primary tumor is HER2-positive. MET is a tyrosine kinase receptor in the membrane surface, and HGF is the main ligand of MET. Although rilotumumab (an anti-HGF antibody) and onartuzumab (an anti-MET antibody) have not been found to confer survival benefit in non-small cell lung or gastric cancers [23–25], rilotumumab reportedly showed survival benefit when used with panitumumab in a phase II trial [26]. MET-positive cancer shows better results than MET-negative cancer when treated with MET inhibitor [27]: MET expression is regarded as a useful biomarker of anti-MET treatment. According to our data, *MET* and *HGF* gene expression in primary CRCs and their metastases is significantly correlated, which is helpful regarding choice of treatment for liver metastases.

Several published studies have reported that the right-sided colon more frequently has *KRAS* and *BRAF* mutations [28–31]. Although we did not analyze *BRAF* mutational status, our data showed a tendency to higher frequency of *KRAS* exon 2 mutation; however, this was not statistically significant because of the small sample size. Maus et al. reported that *BRAF* mutations are more common in right-sided colon, suggested that the predilection of *BRAF* mutation for the right-sided colon partly reflects the high microsatellite instability in this region because such tumors more frequently have *BRAF* mutations [28]. Recently, Guinney et al. reported four consensus molecular subtypes in colorectal cancer, one of which was characterized by hypermutation, microsatellite instability and strong immune activation and comprised 14 % of colorectal cancers [32]. Pembrolizumab, an anti-programmed death 1 (PD-1) immune checkpoint inhibitor, was recently reported to induce remarkable responses in microsatellite unstable colorectal cancers [33]. These data indicate that right-sided colon cancer has more potential to respond to PD-1 inhibitors, in contrast with data indicating that left-sided colon cancer may be more likely to respond to anti-EGFR agents. Tumor location may soon become an important factor when deciding treatment strategy.

## Conclusions

In summary, our data show that *PTEN* mRNA expression differs between left- and right-sided CRCs. We also demonstrated significant correlations between expression of 14 of 19 signal transduction genes in primary tumors and their liver metastases. These data will help in determining why the effects of anti-EGFR antibody differ according to tumor location.

## Abbreviations

CRC, colorectal cancer; CT, threshold cycle; FFPE, formalin-fixed paraffin-embedded; IHC, immunohistochemistry; mCRC, metastatic colorectal cancer; PCR, polymerase chain reaction; PD-1, programmed death 1

## References

[1] Cunningham D, Humblet Y, Siena S, Khayat D, Bleiberg H, Santoro A (2004). Cetuximab monotherapy and cetuximab plus irinotecan in irinotecan-refractory metastatic colorectal cancer. N Engl J Med.

[2] Van Cutsem E, Peeters M, Siena S, Humblet Y, Hendlisz A, Neyns B (2007). Open-label phase III trial of panitumumab plus best supportive care compared with best supportive care alone in patients with chemotherapy-refractory metastatic colorectal cancer. J Clin Oncol.

[3] Brule SY, Jonker DJ, Karapetis CS, O’Callaghan CJ, Moore MJ, Wong R (2015). Location of colon cancer (right-sided versus left-sided) as a prognostic factor and a predictor of benefit from cetuximab in NCIC CO.17.. Eur J Cancer.

[4] von Einem JC, Heinemann V, von Weikersthal LF, Vehling-Kaiser U, Stauch M, Hass HG (2014). Left-sided primary tumors are associated with favorable prognosis in patients with KRAS codon 12/13 wild-type metastatic colorectal cancer treated with cetuximab plus chemotherapy: an analysis of the AIO KRK-0104 trial. J Cancer Res Clin Oncol.

[5] Bufill JA (1990). Colorectal cancer: evidence for distinct genetic categories based on proximal or distal tumor location. Ann Intern Med.

[6] Kaneko Y, Kuramochi H, Nakajima G, Inoue Y, Yamamoto M (2014). Degraded DNA may induce discordance of KRAS status between primary colorectal cancer and corresponding liver metastases. Int J Clin Oncol.

[7] Heinemann V, Modest DP, von Weikersthal LF, Decker T, Kiani A (2014). Gender and tumor location as predictors for efficacy: Influence on endpoints in first-line treatment with FOLFIRI in combination with cetuximab or bevacizumab in the AIO KRK 0306 (FIRE3) trial. J Clin Oncol.

[8] Loupakis F, Yang D, Yau L, Feng S, Cremolini C, Zhang W, et al. Primary tumor location as a prognostic factor in metastatic colorectal cancer. J Natl Cancer Inst. 2015;107(3). doi:10.1093/jnci/dju427. 10.1093/jnci/dju427 PMC4565528 25713148

[9] Besson A, Robbins SM, Yong VW (1999). PTEN/MMAC1/TEP1 in signal transduction and tumorigenesis. Eur J Biochem/FEBS.

[10] Goel A, Arnold CN, Niedzwiecki D, Carethers JM, Dowell JM, Wasserman L (2004). Frequent inactivation of PTEN by promoter hypermethylation in microsatellite instability-high sporadic colorectal cancers. Cancer Res.

[11] Johnson SM, Gulhati P, Rampy BA, Han Y, Rychahou PG, Doan HQ (2010). Novel expression patterns of PI3K/Akt/mTOR signaling pathway components in colorectal cancer. J Am Coll Surg.

[12] Day FL, Jorissen RN, Lipton L, Mouradov D, Sakthianandeswaren A, Christie M (2013). PIK3CA and PTEN gene and exon mutation-specific clinicopathologic and molecular associations in colorectal cancer. Clin Cancer Res.

[13] Lan YT, Jen-Kou L, Lin CH, Yang SH, Lin CC, Wang HS (2015). Mutations in the RAS and PI3K pathways are associated with metastatic location in colorectal cancers. J Surg Oncol.

[14] Sood A, McClain D, Maitra R, Basu-Mallick A, Seetharam R, Kaubisch A (2012). PTEN gene expression and mutations in the PIK3CA gene as predictors of clinical benefit to anti-epidermal growth factor receptor antibody therapy in patients with KRAS wild-type metastatic colorectal cancer. Clin Colorectal Cancer.

[15] Frayling IM, Bodmer WF, Tomlinson IP (1997). Allele loss in colorectal cancer at the Cowden disease/juvenile polyposis locus on 10q. Cancer Genet Cytogenet.

[16] Razis E, Pentheroudakis G, Rigakos G, Bobos M, Kouvatseas G, Tzaida O (2014). EGFR gene gain and PTEN protein expression are favorable prognostic factors in patients with KRAS wild-type metastatic colorectal cancer treated with cetuximab. J Cancer Res Clin Oncol.

[17] Loupakis F, Pollina L, Stasi I, Ruzzo A, Scartozzi M, Santini D (2009). PTEN expression and KRAS mutations on primary tumors and metastases in the prediction of benefit from cetuximab plus irinotecan for patients with metastatic colorectal cancer. J Clin Oncol.

[18] Karapetis CS, Jonker D, Daneshmand M, Hanson JE, O’Callaghan CJ, Marginean C (2014). PIK3CA, BRAF, and PTEN status and benefit from cetuximab in the treatment of advanced colorectal cancer--results from NCIC CTG/AGITG CO.17.. Clin Cancer Res.

[19] Prenen H, De Schutter J, Jacobs B, De Roock W, Biesmans B, Claes B (2009). PIK3CA mutations are not a major determinant of resistance to the epidermal growth factor receptor inhibitor cetuximab in metastatic colorectal cancer. Clin Cancer Res.

[20] Mao C, Wu XY, Yang ZY, Threapleton DE, Yuan JQ, Yu YY (2015). Concordant analysis of KRAS, BRAF, PIK3CA mutations, and PTEN expression between primary colorectal cancer and matched metastases. Sci Rep.

[21] Hocking C, Hardingham JE, Broadbridge V, Wrin J, Townsend AR, Tebbutt N (2014). Can we accurately report PTEN status in advanced colorectal cancer?. BMC Cancer.

[22] Siena S, Sartore-Bianchi A, Lonardi S, Trusolino L, Martino C, Bencardino K, et al. Trastuzumab and lapatinib in HER2-amplified metastatic colorectal cancer patients (mCRC): The HERACLES trial. J Clin Oncol. 2015;33(suppl;abstr 3508).

[23] Cunningham D, Tebbutt N, Davidenko I, Murad A, Al-Batran S, Ilson D, et al. Phase III, randomized, double-blind, multicenter, placebo (P)-controlled trial of rilotumumab (R) plus epirubicin, cisplatin and capecitabine (ECX) as first-line therapy in patients (pts) with advanced MET-positive (pos) gastric or gastroesophageal junction (G/GEJ) cancer: RILOMET-1 study. J Clin Oncol. 2015;33(suppl;abstr 4000).

[24] Shah M, Bang Y, Lordick F, Tabernero J, Chen M, Hack S. METGastric: A phase III study of onartuzumab plus mFOLFOX6 in patients with metastatic HER2-negative (HER2-) and MET-positive (MET+) adenocarcinoma of the stomach or gastroesophageal junction (GEC). J Clin Oncol. 2015;33(suppl:abstr 4012).

[25] Spigel D, Edelman M, O’Byme K, Paz-Ares L, Shames D, Yu W. Onartuzumab plus erlotinib versus erlotinib in previously treated stage IIIb or IV NSCLC: Results from the pivotal phase III randomized, multicenter, placebo-controlled METLung (OAM4971g) global trial. J Clin Oncol. 2014;52(s):Suppl;abstr 8000. 10.1200/JCO.2016.69.2160 27937096

[26] Van Cutsem E, Eng C, Nowara E, Swieboda-Sadlej A, Tebbutt NC, Mitchell E (2014). Randomized phase Ib/II trial of rilotumumab or ganitumab with panitumumab versus panitumumab alone in patients with wild-type KRAS metastatic colorectal cancer. Clin Cancer Res.

[27] Koeppen H, Yu W, Zha J, Pandita A, Penuel E, Rangell L (2014). Biomarker analyses from a placebo-controlled phase II study evaluating erlotinib+/−onartuzumab in advanced non-small cell lung cancer: MET expression levels are predictive of patient benefit. Clin Cancer Res.

[28] Maus MK, Hanna DL, Stephens CL, Astrow SH, Yang D, Grimminger PP (2015). Distinct gene expression profiles of proximal and distal colorectal cancer: implications for cytotoxic and targeted therapy. Pharmacogenomics J.

[29] Rosty C, Young JP, Walsh MD, Clendenning M, Walters RJ, Pearson S (2013). Colorectal carcinomas with KRAS mutation are associated with distinctive morphological and molecular features. Mod Pathol.

[30] Yamauchi M, Morikawa T, Kuchiba A, Imamura Y, Qian ZR, Nishihara R (2012). Assessment of colorectal cancer molecular features along bowel subsites challenges the conception of distinct dichotomy of proximal versus distal colorectum. Gut.

[31] Samowitz WS, Curtin K, Schaffer D, Robertson M, Leppert M, Slattery ML (2000). Relationship of Ki-ras mutations in colon cancers to tumor location, stage, and survival: a population-based study. Cancer Epidemiol Biomarkers Prev.

[32] Guinney J, Dienstmann R, Wang X, de Reynies A, Schlicker A, Soneson C (2015). The consensus molecular subtypes of colorectal cancer. Nat Med.

[33] Le DT, Uram JN, Wang H, Bartlett BR, Kemberling H, Eyring AD (2015). PD-1 blockade in tumors with mismatch-repair deficiency. N Engl J Med.

